# Correlation of Viral Load With the Biochemical and Hematological Profiles of COVID-19 Patients in Al Buraimi Hospital, Sultanate of Oman: A Cross-Sectional Study

**DOI:** 10.7759/cureus.35228

**Published:** 2023-02-20

**Authors:** Eida Al Badi, Intisar Al Shukri, Samira Al Mahruqi

**Affiliations:** 1 Microbiology, Al Buraimi Hospital, Al Buraimi, OMN; 2 Virology, Central Public Health Laboratory, Directorate General for Disease Surveillance and Control, Muscat, OMN; 3 Virology, Oman Country Office, Global Health, The Association of Public Health Laboratories, Muscat, OMN

**Keywords:** rt-pcr laboratory markers, viral load and lab markers, sars-cov-2 diagnosis, ct value, covid-19 pandemic

## Abstract

Background

Rapid identification of COVID-19 is crucial during the pandemic for the treatment and management of patients. Thus, early diagnosis of the disease using laboratory parameters can help in the rapid management of infected patients. This study aimed to investigate the correlation of viral load with hematological and biochemical parameters. This will ultimately help physicians to better understand the dynamics of this novel virus and aid in the management of patients.

Methodology

Laboratory confirmation of SARS-CoV-2 was performed by reverse transcription-polymerase chain reaction (RT-PCR) at the Al-Buraimi Hospital Laboratory Department using oropharyngeal and nasopharyngeal swabs. Positive cases were collected from July 2020 to January 2021 to be enrolled in this study.

Results

In this study, 264 confirmed positive patients were included initially and divided into three groups according to their cycle threshold (Ct) values obtained by PCR. Out of the total 264 patients, 174 (65.9%) were male, while 90 (34.1%) were female. However, the final sample was only 253 patients who met the inclusion criteria. With regard to Ct values, the study population was divided into the following three groups: Group 1 with Ct values of 9-20 (n = 87; 34.4%), group 2 with Ct values of 21-30 (n = 122; 47.8%), and group 3 with Ct values of 31-42 (n = 44; 17.4%).

Conclusions

We found that the proportion of male patients infected with COVID-19 was higher compared to females. In addition, the highest incidence was among patients in the age group of 51-70 years. The ferritin and alanine transaminase levels were highest in the initial stage of the infection (group 1) and decreased at the recovery stage. However, neutrophil, lymphocyte, alkaline phosphatase, and C-reactive protein showed an increasing trend from high viral load groups to low viral load groups. The values of the rest of the parameters, such as albumin, total bilirubin, lactate dehydrogenase, and D-dimer, were slightly higher in the initial stage of the infection but the decreasing trend was low; therefore, they were not considered helpful in predicting the disease severity reflected by their Ct value in the three different groups.

## Introduction

Coronavirus disease 2019 (COVID-19) is a novel disease caused by severe acute respiratory syndrome coronavirus 2 (SARS-CoV-2). SARS-CoV-2 infection has an extensive range of clinical manifestations varying from asymptomatic to symptomatic consisting of respiratory symptoms, fever, shortness of breath, cough, dyspnea, and viral pneumonia. In severe cases, it can lead to severe acute respiratory syndrome, heart failure, and renal failure [1]. Some of these symptoms are the predominant cause of COVID-19-associated death [2]. The major complications of COVID-19 are pneumonia and acute respiratory distress which elicit immune responses causing massive uncontrolled inflammation and tissue damage [2]. Furthermore, the multisystem inflammatory syndrome in children (MIS-C) has been confirmed to be associated with SARS-CoV-2 infection [3]. Some studies have shown that the severity of the disease is related to viral load in terms of hematological and biochemical parameters while others have reported no direct correlation [4,5].

Several common laboratory parameters act as predictors of the severity of COVID-19 infection and play an important role in managing the disease prognosis and prediction of treatment decisions [6]. These common parameters include albumin, C-reactive protein (CRP), alanine aminotransferase (ALT), D-dimer, ferritin, total bilirubin (TB), alkaline phosphatase (ALP), lactate dehydrogenase (LDH), neutrophil, and lymphocytes.

In this study, the direct indicator of COVID-19, the cycle threshold (Ct) value, has been correlated with the laboratory biochemical and hematological parameters in confirmed COVID-19 patients.

## Materials and methods

Laboratory confirmation of SARS-CoV-2 was performed by reverse transcription-polymerase chain reaction (RT-PCR) at Al Buraimi Hospital Laboratory Department, which is a bio-safety level (BSL-II) laboratory using the BD MAX system (MAX SARS-CoV-2 assay; Becton, Dickinson and Company, BD Life Sciences-Integrated Diagnostic Solutions, Sparks, MD, USA), which is a fully integrated bench-top molecular diagnostic system. The biochemical and hematological parameters were performed using the COBAS C311 chemistry analyzer (Roche-Hitachi, France) and CELL DYN RUBY (Automated Haematology Analyzer, Abbott Diagnostics, USA), respectively. The study protocol was approved by the Research Ethical Review and Approval Committee, Al Buraimi Governorate (proposal ID: MoH/CSR/21/25224).

Study period, study design, and inclusion and exclusion criteria

This cross-sectional, retrospective study was conducted on COVID-19 PCR-positive patients (n = 264) who were admitted to Al Buraimi Hospital from July 2020 to January 2021. The inclusion and exclusion criteria are listed in Table 1.

**Table 1:** Inclusion and exclusion criteria of this cross-sectional, retrospective study. COVID-19 = coronavirus disease 2019; PCR = polymerase chain reaction

Inclusion criteria	Exclusion criteria
Confirmed PCR-positive patients (Ct value ≤42 for COVID-19) using nasopharyngeal and oropharyngeal swabs collected in viral transport media	Non-admitted patients and suspected COVID-19 patients with PCR-negative reports
Correctly labeled and barcoded vials with proper sample quality	Improperly labeled vials and grossly mucoid and bloody samples
Serum samples taken from COVID-19 PCR-positive patients (n = 264) were used to analyze biochemical lab parameters	Positive COVID-19 reports from dead patients without previous laboratory investigations

Statistical analysis

The data were retrieved from the Electronic System (NEHR Al-Shifa) at Al Buraimi Hospital. The study population was divided into the following four categories: above 70 years, between 51 and 70 years, between 36 and 50 years, and up to 35 years. Spearman correlational analysis between the groups and the Ct values were computed using SPSS version 24 (IBM Corp., Armonk, NY, USA). A two-sided p-value <0.05 was considered statistically significant. Descriptive statistics including the mean and standard deviation (SD) of all biochemical and hematological parameters (albumin, ALT, TB, CRP, LDH, ferritin, D-dimer, neutrophil, and lymphocytes) were computed.

## Results

Of the 264 patients, 174 (65.9%) were male, while 90 (34.1%) were female. However, the final sample was 253 patients who met the inclusion criteria. In this study, 253 confirmed COVID-19-positive patients were included and divided into three groups according to their Ct values obtained by PCR. Group 1 had Ct values of 9-20 (n = 87; 34.4%), group 2 had Ct values ranging from 21 to 30 (n = 122; 47.8%), and group 3 had Ct values ranging from 31 to 42 (n = 44; 17.4%). The majority of the patients in this study were in the age group of 51-70 years (32.2%), followed by 29.5% of patients in the age group of 36-50 years, and 15.2% of patients in the age group of >70 years (15.2%).

Group 1 (Ct values 9-20, N = 87)

Computed means and SDs for each of the biochemical parameters in group one were albumin (n = 83; mean = 36.17 ± 0.57; SD = 5.16), ALT (n = 83; mean = 62.88 ± 22.89; SD = 208.53) TB (n = 83; mean = 10.72 ± 1.33; SD = 12.14), CRP (n = 87; mean = 86.85 ± 8.53; SD = 79.58), LDH (n = 65; mean = 421.01 ± 51.81; SD = 449.99), ferritin (n = 86; mean = 2,160.95 ± 578.74; SD = 6,294.46), D-dimer (n = 45; mean = 3.27 ± 1.20; SD = 8.05), ALP (n = 83; mean = 79.15 ± 3.87; SD = 35.21), neutrophil (n = 87; mean = 4.90 ± 0.41 SD = 3.78), and lymphocytes (n = 87; mean = 1.39 ± 0.10; SD = 0.96).

Group 2 (Ct values 21-30, N = 122)

The means and SDs for each of the biochemical parameters in group two were also computed and results showed albumin (n = 115; mean = 36.36 ± 0.48; SD = 5.10), ALT (n = 115; mean = 47.22 ± 6.49; SD = 69.34), TB (n = 83; mean = 10.73 ± 1.33; SD = 12.15), CRP (n = 121; mean = 96.08 ± 7.32; SD = 80.22), LDH (n = 93; mean = 396.57 ± 17.02; SD = 163.28), ferritin (n = 121; mean = 1,276.46 ± 230.21; SD = 2,521.82), D-dimer (n = 56; mean = 1.68 ± 0.42; SD = 3.12), ALP (n = 115; mean = 101.85 ± 9.80; SD = 104.68), neutrophil (n = 122; mean = 5.10 ± 0.29; SD = 3.19), and lymphocytes (n = 122; mean = 1.40 ± 0.10; SD = 1.04).

Group 3 (Ct values 31-42, N = 44)

The means and SDs for each of the biochemical parameters in group three were calculated and results showed albumin (n = 42; mean = 34.65 ± 1.01; SD = 6.55), ALT (n = 42; 33.52 ± 5.90; SD = 38.20), TB (n = 42; 15.12 ± 6.36; SD = 41.23), CRP (n = 44; mean = 104.07 ± 18.52; SD = 122.86), LDH (n = 30; mean = 305.93 ± 34.73; SD = 190.25), ferritin (n = 40; mean = 772.80 ± 145.03; SD = 917.27), D-dimer (n = 14; mean = 2.47 ± 0.91; SD = 3.42), ALP (n = 43; mean = 158.33 ± 66.04; SD = 433.03), neutrophil (n = 44; mean = 7.61 ± 1.00; SD = 6.66), and lymphocytes (n = 44; mean = 1.94 ± 0.16; SD = 1.03).

Table 2 and Table 3 present the correlation of the biochemical and hematological profiles with Ct values in the three groups.

**Table 2:** Ct values in the three groups. Ct = cycle threshold

	Group 1	Group 2	Group 3
Ct value	9–12	21–30	31–42
Mean Ct value	1.05 ± 0.023	2.13 ± 0.165	2.96 ± 0.031
N (253)	87	122	44
Age (year)	52.83 ± 2.01	50.30 ± 1.47	46.20 ± 3.33
Male (%)	52 (60.47)	78 (63.93)	24 (53.33)
Female (%)	34 (39.53)	44 (36.07)	21 (46.67)

**Table 3:** The correlation of the biochemical and hematological profiles with Ct values in the three groups. Ct = cycle threshold; ALT = alanine aminotransferase; TB = total bilirubin; CRP = C-reactive protein; LDH = lactate dehydrogenase; ALP = alkaline phosphatase

Biochemical and hematological profiles	Group 1	Group 2	Group 3	P-value
Albumin	36.17 ± 0.57 (0.383)	36.36 ± 0.48 (0.124)	34.66 ± 1.01 (0.442)	0.109
ALT	62.88 ± 22.89 (0.882)	47.22 ± 6.9 (0.198)	33.53 ± 5.90 (0.171)	0.206
TB	10.73 ± 1.33 (0.876)	10.73 ± 1.33 (0.917)	15.12 ± 6.36 (0.997)	0.340
CRP	86.86 ± 8.53 (0.537)	96.08 ± 7.32 (0.842)	104.08 ± 18.52 (0.751)	0.515
LDH	421.02 ± 51.81 (0.425)	396.57 ± 17.02 (0.734)	305.93 ± 34.73 (0.557)	0.123
Ferritin	2,160.96 ± 578.74 (0.677)	1,276.46 ± 230.21 (0.667)	772.80 ± 145.03 (0.097)	0.060
D-dimer	3.27 ± 1.20 (0.850)	1.68 ± 0.42 (0.709)	2.47 ± 0.91 (0.786)	0.836
ALP	79.16 ± 3.87 (0.048)	101.85 ± 9.80 (0.366)	158.33 ± 66.04 (0.744)	0.054
Neutrophil	4.90 ± 0.41 (0.732)	5.06 ± 0.29(0.907)	7.61 ± 1.00 (0.333)	0.004
Lymphocytes	1.40 ± 0.10 (0.508)	1.40 ± 0.10 (0.94)	1.94 ± 0.16 (0.357)	0.010

## Discussion

The outbreak of the COVID-19 pandemic raises concern regarding the importance of laboratory markers in detecting the disease. In this regard, Ct values have been associated with various biochemical markers. Rabaan et al. (2021) found that biochemical parameters, such as ALT, bilirubin, LDH, CRP, and albumin, play a crucial role in predicting RT-PCR-positive COVID-19 cases [7]. In this study, albumin, ALT, TB, CRP, LDH, ferritin, D-dimer, neutrophil, and lymphocytes were analyzed.

**Figure 1:**
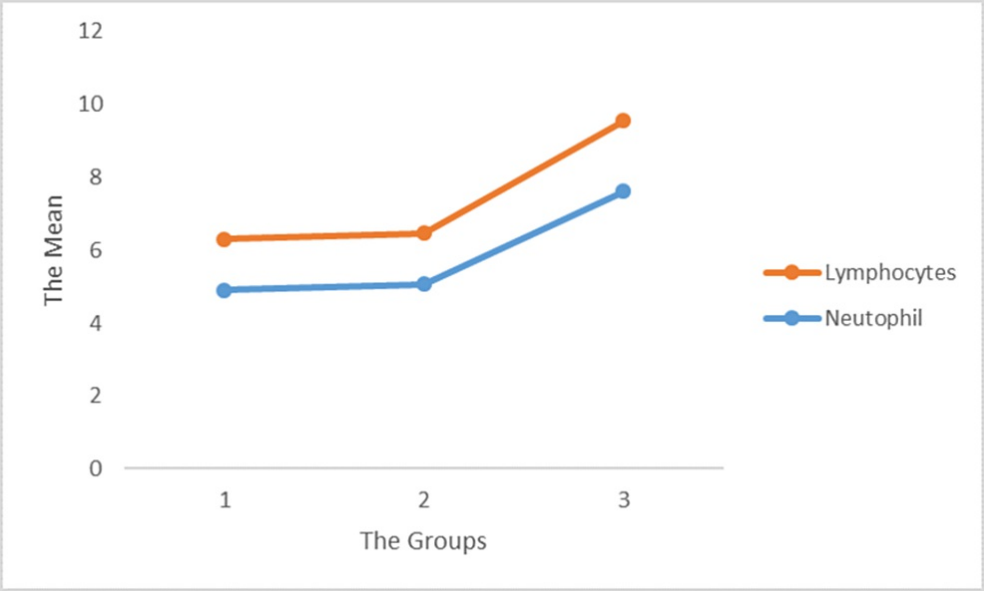
Correlation of lymphocytes and neutrophils with the viral load in the different groups.

In the immune system, neutrophils are essential for fighting infection and healing wounds. Cheng et al. (2020) determined that the number of circulating neutrophils increased dramatically as COVID-19 progressed [8]. Not only was the increase in neutrophils found in the bloodstream but also in the lung tissue. Wang (2020) explained that the increased infiltration of immature and dysfunctional neutrophils was attributed to the weak immune response in the lungs of critically ill patients [9]. Therefore, SARS-CoV-2 infection increases the release of extracellular neutrophil traps that lead to tissue damage. Another study by Liu et al. (2020) found that the neutrophil-to-lymphocyte ratio (NLR) was an independent risk factor for severe disease in patients with COVID-19 [10]. In this study, neutrophils (p = 0.004) and lymphocytes (p = 0.01) increased in all three groups. Both parameters showed an increasing trend from high viral load groups to low viral load groups (Figure 1). These results showed that in all patients in the three groups, the presence of the virus in the body increased the levels of neutrophils and lymphocytes. Additionally, this finding demonstrated that as the oxygen levels of COVID-19 patients decreased, their neutrophil count increased dramatically, confirming the presence of infection in the body.

**Figure 2:**
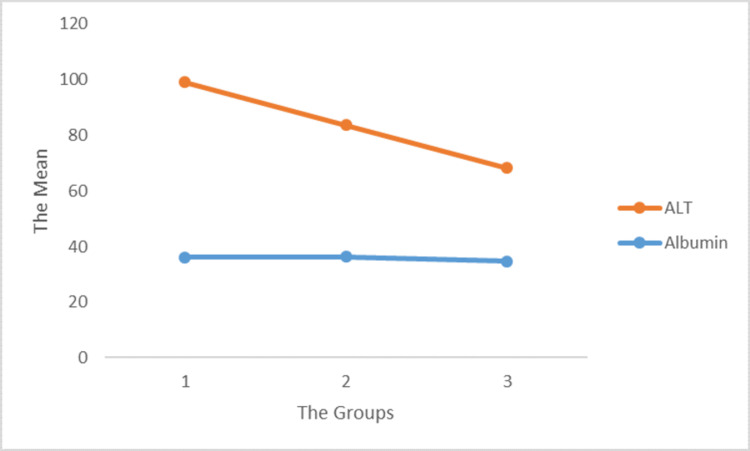
Correlation of ALT and albumin with the viral load in the different groups. ALT = alanine aminotransferase

ALT is an enzyme necessary for the detection of liver abnormalities in COVID-19 patients. A meta-analysis of 13 studies showed that the combined mean of albumin levels was 38.04 g/L (34-54 g/L), with significant heterogeneity [11]. Albumin levels are important in identifying patients infected with SARS-CoV-2 with an inflammatory process. In this study, albumin and ALT decreased in all three groups. In terms of significance level, i.e., albumin (p = 0.109) and ALT (p = 0.206), the change in trend was not significant in predicting the severity of COVID infection, which is inherent. Because higher levels of ALT and albumin have been observed in patients with COVID-19, tests for liver abnormalities can be considered indicators of infection [11]. However, the p-value of the correlation was insignificant, which makes it trivial to consider (Figure 2).

In COVID-19, ferritin is an inflammatory biomarker that acts as an acute-phase reactant reflecting the extent of chronic and acute inflammatory responses in the body [12]. Vargas-Vargas and Cortés-Rojo (2020) demonstrated that ferritin is a key mediator of immune dysregulation, especially in extreme hyperferritinemia [12]. This is enhanced by the direct effects of immunosuppression and pro-inflammation, which may lead to a cytokine storm with fatal outcomes for COVID-19-infected patients. Therefore, the severity of COVID-19 depends on this cytokine storm syndrome [12]. In this study, ferritin levels in all three groups showed a decreasing trend. Moreover, in the three groups, the p-value (0.060) was not statistically significant (p > 0.05). Furthermore, the three study groups had the highest average ferritin levels. However, the mean ferritin value was highest in group 1 patients, with Ct values ranging ​​between 9 and 20. LDH is an enzyme that converts pyruvate to lactate in glucose metabolism. LDH secretion is caused by cell membrane necrosis, a sign of lung injury or viral infection caused by SARS-CoV-2 [13]. There is good evidence that LDH levels are associated with COVID-19 disease severity [14]. For example, Guan et al. (2020), in a study of 1,099 patients, found sufficient evidence that the extent of tissue damage was associated with increased levels of LDH [15]. CT scans showed that higher levels of LDH correlated with the severity of pneumonia. Zhang et al. (2018) showed that there was a positive correlation between chest CT scores and serum CRP, LDH, and ferritin, concluding that CT scores are a good marker of the extent of systemic inflammation [16].

**Figure 3:**
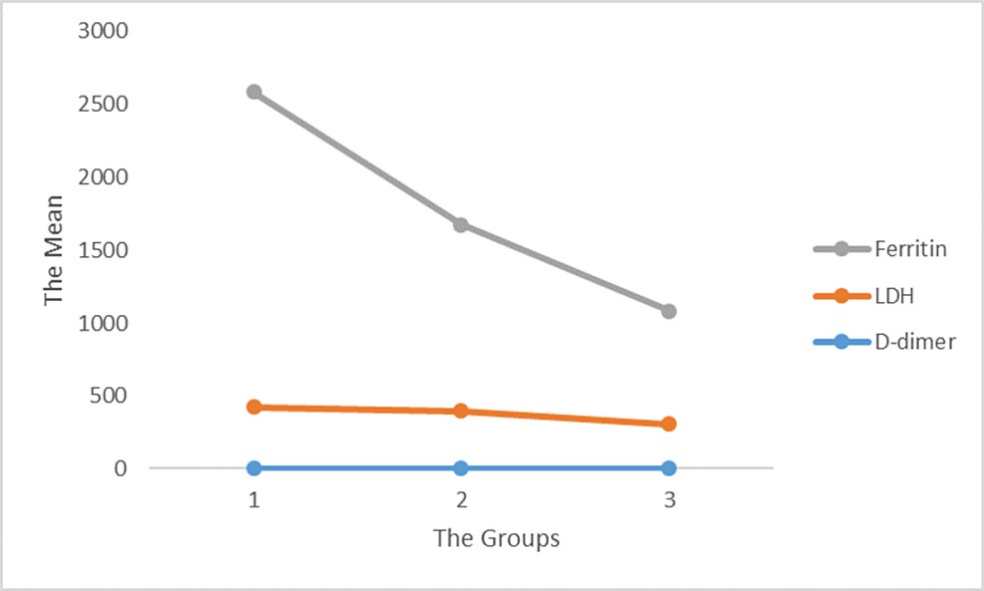
Correlation of ferritin, LDH, and D-dimer with the viral load in the different groups. LDH = lactate dehydrogenase

D-dimers result from the cleavage of cross-linked fibrin, and its increased levels indicate activation of fibrinolysis and coagulation [17]. Prior studies have linked COVID-19 with abnormal hemostasis with elevated levels of D-dimers [18]. Similarly, a retrospective cohort study involving 191 patients determined that D-dimer levels >1.0 μg/mL (p = 0.0033) were associated with increased mortality in COVID-19 patients. Additionally, they reported that a level of 2.0 μg/mL or higher on admission serves as a baseline to predict in-hospital mortality from COVID-19 [19]. Moreover, previous studies have shown that at least 90% of hospitalized patients with pneumonia report increased coagulation activity characterized by rising levels of D-dimer [17,18]. In this study, D-dimer and ferritin showed a decreasing trend across the three groups which illustrates that as the viral load decreases the levels of the latest markers also decrease. However, there was no defined trend in LDH across the three groups (Figure 3).

CRP is a plasma protein produced by the liver and induced by various inflammatory mediators including interleukin-6 [20]. Gong et al. (2020) reported that CRP can be used clinically as a biomarker for inflammatory diseases such as COVID-19 [20]. Additionally, increased CRP levels were shown to be associated with increased disease severity [21]. A retrospective cohort study by Liu et al. (2020) showed an increased likelihood of progression to severe COVID-19 in patients with CRP levels >41.8 mg/L [10]. Both studies considered CRP levels as a strong indicator of the presence and severity of COVID-19. In this study, there was an increase in the mean values of CRP across the three groups, indicating that CRP levels increase with disease prognosis. However, the p-value for CRP did not reach a significant level (p < 0.05).

**Figure 4:**
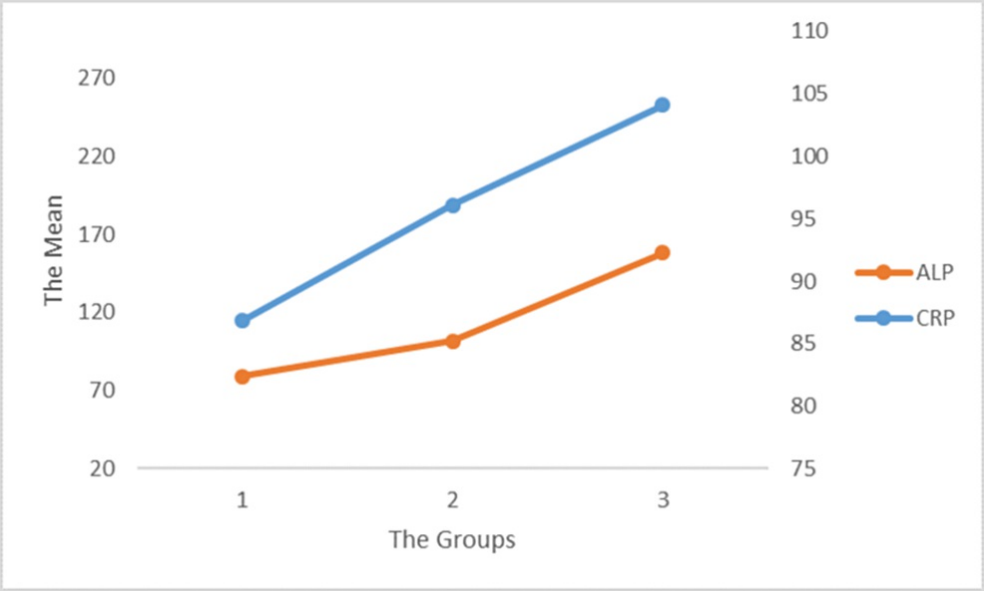
Correlation of ALP and CRP with the viral load in the different groups. ALP = alkaline phosphatase; CRP = C-reactive protein

ALP is an enzyme present in different parts of the body. ALP levels among patients in the three groups were elevated, indicating that patients with lower Ct values witnessed a significant increase in ALP, denoting the presence of severe infection or the presence of disease (p = 0.054). Liver abnormalities have been found in patients with low Ct values, and a high ALP value indicates severe COVID infection [22]. Doctors and healthcare professionals can use ALP values to diagnose the presence of an infection in the body. In this study, a p-value of 0.34 was seen for TB levels, which was above the significance level. Therefore, it is not reasonable to consider these as indicators (Figure 4).

Summary of the findings

The findings of the current study suggest that the proportion of male patients admitted with COVID-19 was higher compared to females. In addition, the highest incidence was among patients in the age group of 51-70 years. Group 1 patients had the lowest Ct value, which showed that they had the highest viral load. Furthermore, the highest viral load was associated with greater significant ALP changes, indicating more severe liver damage among COVID-19-infected patients. As the presence of the COVID-19 virus hampers the overall functioning of the body, the patients had witnessed abnormal liver functions, which negatively affected their ALP levels [23]. Moreover, ferritin and ALT levels were the highest in the initial stage of the infection and decreased during the recovery stage. The values of the rest of the parameters, such as albumin, TB, LDH, and D-dimer, were slightly higher in the initial stage of the infection but the decreasing trend was low; hence, they did not have a significant impact on the Ct value and COVID-19 severity. In group 2, with increased Ct values, the viral load decreased, which lowered the influence of the COVID-19 virus on the admitted patients. Finally, for group 3, the highest Ct value showed that patients’ viral load was very low, highlighting a lower chance of developing COVID-19 complicated symptoms. The disease progression in this group indicated that they were in the recovery stage. The possible reason for the absence of significant association in some of the parameters of our study was that most of the study samples were in group 2 where the viral load was slightly low compared to group 1.

Limitations and future work

This study suffers from the limitation of non-responses as data on some lab parameters were not available due to a sudden lack of reagents, which likely reduced the overall reliability of the research findings. Furthermore, the study’s sample size was small as data were gathered from only 253 patients who mostly belonged to the second stage of infection and were under treatment, according to Ct values. To improve the quality of results in future studies, regression analysis can be applied to further examine the extent of association between the independent and dependent variables. Moreover, more precise and complete data including clinical manifestation of the study patients will improve the quality of research results and relate it more to the patient’s clinical presentations.

## Conclusions

The study showed that some laboratory markers can help in determining disease severity, such as ferritin and ALT, which were the highest in the initial stage of COVID-19 infection and decreased during the recovery stage. However, neutrophil, lymphocytes, ALP, and CRP levels showed an increasing trend from high viral load groups to low viral load groups. The rest of the parameters, such as albumin, TB, LDH, and D-dimer, were slightly higher in the initial stage of the infection but the decreasing trend was low; therefore, they cannot be considered helpful in predicting the disease severity.
